# Mechanisms underlying firing in healthy and sick human motoneurons

**DOI:** 10.3389/fnhum.2015.00174

**Published:** 2015-03-30

**Authors:** Maria Piotrkiewicz, Parveen N. S. Bawa, Annie Schmied

**Affiliations:** ^1^Engineering of Nervous and Muscular System, Nałęcz Institute of Biocybernetics and Biomedical Engineering, Polish Academy of SciencesWarsaw, Poland; ^2^Department of Biomedical Physiology and Kinesiology, Simon Fraser UniversityBurnaby, BC, Canada; ^3^Centre National de la Recherche Scientifique, Plasticité et Pathophysiologie du Mouvement, Institut de Neuroscience de la Timone, University Aix MarseillesMarseille, France

**Keywords:** human motoneuron discharge mechanisms, motor unit, afterhyperpolarization and delayed depolarization, double and triple discharges, recruitment and rate coding, axon and peripheral afferents, excitability, motoneuron pathology

In an address to the British Association for the Advancement of Science in Cambridge, Professor Sherrington introduced the terms “*motor neurone*” and “*the final common path*,” the latter term implying that all motor commands converge onto the motoneuron which integrates the incoming information and passes the net information to the muscle for contraction (Sherrington, 1904). The relative ease of access of the spinal motoneuron made it feasible to set up techniques for investigating the physiological, biophysical and molecular properties of these neurons. It became the most investigated neuron of the CNS in the twentieth century and the information gained from studies on motoneurons formed the basis for examining the other neurons of the CNS. Since the compound action potential of a muscle unit is strictly related one-to-one to the action potential arriving from the innervating motoneuron, the statistical analysis of muscle unit action potentials provides an investigator with an elegant way to probe the properties of motoneurons in behaving humans. In the following review the terms *motoneuron* and *motor unit* might be used interchangeably. Different aspects of human motoneuron investigations in health and disease are presented in 16 articles of this topic which are summarized below.

An increase in the net excitatory synaptic input to the motoneuron pool results in an increase in the level of muscle contraction by recruitment of additional motor units (MUs) and an increase in firing rates of the already recruited units (Milner-Brown et al., 1973; Henneman et al., 1974). The principle of orderly recruitment of motoneurons by size was originally proposed by Henneman (1957) but was later questioned by other researchers presenting examples of selective, rather than orderly recruitment (e.g., Smith et al., 1980). These controversies are assessed by Bawa et al. (2014), and the opinion unifying the concept of orderly recruitment is presented. In humans, increases in firing rates of motor units have been shown to follow the “onion skin” pattern at lower levels of contraction, meaning that the lower-threshold motor units discharge with higher rates than higher-threshold ones. However, studies performed on the whole range of muscle forces indicated that for higher force levels the motor unit firing rate follows a “reverse onion skin” pattern. Hu et al. (2014) decided to approach this problem using small surface electrodes and step increases in force instead of the “ramp and hold” protocols used by previous authors. They showed that the “onion skin” pattern was preserved until 15% of maximal voluntary contraction, and from their results predict this pattern to be valid for the whole range of muscle forces, which is not supported by the previous published works. However, the reported rate saturation of the MUs discharging with higher rates implies that at the higher forces the “reverse onion skin” pattern may be expected. In another paper, Duchateau and Baudry (2014) show that during ballistic contractions the maximal discharge rates are higher than those observed in ramp contractions. It should be noted, however, that during ballistic contractions one deals with instantaneous rates, while during ramp and hold contractions one refers to tonic firing rates defined as the average over 1 s. One cannot compare maximal rates during the two patterns of contraction. The authors also suggest that the maximal rate of force development is determined by maximal instantaneous firing rate of the motoneurons confirming earlier work on reduced cat preparations using intracellular current injections (Baldissera et al., 1982). For information of firing rates in older adults, the maximal firing rates have been reported to decline (Duchateau and Baudry, 2014; Kallio et al., 2014).

The high instantaneous firing rates have also been observed at low, slower speeds of muscle contractions. They are generated by some motoneurons, which occasionally fire pairs of closely spaced spikes (doublets); each pair is followed by a prolonged post doublet interval suggested to result from the summation of successive afterhyperpolarizations (AHPs). The “true” doublets (Bawa and Calancie, 1983) should be distinguished from the short interspike intervals fired during ballistic contractions. The former are attributed to the delayed depolarization, an intrinsic property of the motoneuron (Kernell, 1964; Calvin, 1973), while the latter are due to the high rate of rise in synaptic inputs (Baldissera et al., 1982). Doublets have been reported by several authors contributing to the present topic. Piotrkiewicz et al. (2013) observed doublets in the soleus muscle where they have never been documented before. Repetitive doublets, where doublet/postdoublet intervals alternate, were first reported by Bawa and Calancie (1983). Kudina and Andreeva (2010) suggested earlier that repetitive doublets resulted from suprathreshold delayed depolarization supported by plateau potentials. In the present article, Kudina and Andreeva (2013) pose the question whether there are common attributes of motoneurons in the spinal cord which can fire repetitive doublets. They suggest a cranio-caudal gradient of the number of motoneurons that can discharge doublets and more frequent repetitive doublets observed in the cervical motoneurons compared to the motoneurons of the lumbar region. Piotrkiewicz and Kuraszkiewicz (2014) investigated the relationship between duration of motoneuron AHP and confirmed an earlier observation by Kernell (1964) that motoneurons with shorter AHPs discharge doublets more easily. Doublets were also mentioned in Søgaard et al. (2014). Triplet discharges have been reported by Piotrkiewicz and Kuraszkiewicz (2014) and the authors speculate on the possible mechanisms to explain this pattern.

Three papers deal with problems of motoneuron excitability. Various methods used to date to test excitability of human motoneuron pools are reviewed by McNeil et al. (2013). While synaptic inputs to a motoneuron pool recruit motoneurons from small to large, electrical stimulation of a muscle nerve recruits motoneurons in reverse order, from larger to smaller axons. It has been acknowledged for some time that the diameter is not the only factor determining axon excitability. In a mixed nerve, sensory and motor fibers have different biophysical properties making sensory afferents more excitable than motor axons of the same size. Human studies of axon excitability have obvious limitations, thus Lorenz and Jones (2014) explored this problem in a rat model. They have shown that several biophysical parameters differ between axons innervating slow soleus and fast tibialis anterior. These mechanisms may underlie the bimodal distribution of axon excitability observed in the tibialis anterior by Kudina and Andreeva (2014). Since the axons of larger motoneurons are more excitable, electrical stimulation used for rehabilitation purposes might only recruit and strengthen the larger motor units and let the small units succumb to atrophy. Dean et al. (2014) have proposed a method to recruit smaller low threshold units using specially tailored electrical stimulation of a mixed nerve. The stimulus consists of a high frequency pulse train with current strength subthreshold for eliciting M or H waves. In soleus, stimulation of the posterior tibial nerve with such parameters has been shown to recruit motor units in an orderly fashion from small to large, and at physiological firing rates thus leading to activation and strengthening of small motor units.

Six papers deal with motoneuron discharge related to pathology. Søgaard et al. (2014) recorded electromyographic activity bilaterally from upper trapezius muscles. They observed a tonic low level motor unit activity and suggested that such activity, whether it is built into the motor program to stabilize the limbs or results from stress of paying attention during computer work, may be the underlying cause of myalgia observed in computer workers. Garland et al. (2014) reviewed stroke-related changes in motor unit discharge characteristics and suggested that residual motor control strategies may remain after stroke. McNulty et al. (2014) investigated motor unit firing characteristics in different muscles of stroke survivors showing that the effects on peak firing rates and their dynamic range differ not only between joints of the upper and lower limbs but also between muscles of different joints of the same limbs. Furthermore, they have shown that motor units on both paretic and non-paretic sides changed after stroke. Confirming the common observation that firing rates on the affected side are lower than normal, they have also shown that motor units from the unaffected side discharge with firing rates higher than normal. The authors conclude that motor unit properties on both sides should be compared to data from age- and sex-matched healthy subjects.

Neuroscientists have used peripheral inputs to investigate motor output and various properties of motoneurons. Yet, when it comes to voluntary control of movement, the peripheral afferents are generally ignored. In their paper describing extensive single motor unit recordings from a deafferented subject, Schmied et al. (2014) demonstrate the importance of peripheral afferents in the control of motoneuron excitability, variability in firing, synchronization and coherence between different motor units. The viability of motoneurons and the dependence of their excitability on peripheral afferents are discussed by Zijdewind et al. (2014). In incomplete spinal cord injured subjects the mean firing rates decrease compared to those in normal subjects. The authors argue that the decrease in firing rates is not due to changes in motoneuron properties resulting from a decrease or complete elimination of descending inputs. These motoneurons are capable of reaching normal discharge rates during spasms. However, the covariation in firing rates among various motor units mentioned above by Hu et al. (2014) is lacking during spasms, which implies that the weight of different inputs onto different motoneurons of a pool might alter in spinal cord injury. This might also apply to persistent inward currents of the members of a pool.

Motoneurons form the final motor path for all the motor commands; an animal without motoneurons would die. Such is the case in amyotrophic lateral sclerosis (ALS), a deadly disease of unknown etiology, which causes selective degeneration of spinal motoneurons and corticomotoneuronal cells (“lower” and “upper” motoneurons, respectively). De Carvalho et al. (2014) review the properties of the upper and lower motoneurons in ALS, aiming to show how changes in the pattern of motor unit firing could help to delineate the underlying pathophysiological disturbance as the disease progresses. However, some of the few single motor unit studies performed in patients with ALS have not been covered in this review. The interested reader may find additional information in Schmied et al. (1999), Attarian et al. (2006, 2008).

The articles collected in this exciting Research Topic cover a broad spectrum of human motoneuron research, from their intrinsic properties such as afterhyperpolarization following delayed depolarization to pathological changes in neuromuscular disorders. We hope that this collection will be equally exciting for the potential readers. Our sincere thanks are expressed to all the authors and reviewers who contributed to this important topic of human neurophysiology.

## Conflict of interest statement

The authors declare that the research was conducted in the absence of any commercial or financial relationships that could be construed as a potential conflict of interest.
